# Rare disease genomics and precision medicine

**DOI:** 10.1186/s44342-024-00032-1

**Published:** 2024-12-03

**Authors:** Juhyeon Hong, Dajun Lee, Ayoung Hwang, Taekeun Kim, Hong-Yeoul Ryu, Jungmin Choi

**Affiliations:** 1 Department of Biomedical Sciences, Korea University College of Medicine, Seoul, 02841 Republic of Korea; 2 School of Life Sciences, BK21 FOUR KNU Creative BioResearch Group, College of Natural Sciences, Kyungpook National University, Daegu, 41566 Republic of Korea

**Keywords:** Rare disease genomics, Big data analytics, Precision medicine

## Abstract

Rare diseases, though individually uncommon, collectively affect millions worldwide. Genomic technologies and big data analytics have revolutionized diagnosing and understanding these conditions. This review explores the role of genomics in rare disease research, the impact of large consortium initiatives, advancements in extensive data analysis, the integration of artificial intelligence (AI) and machine learning (ML), and the therapeutic implications in precision medicine. We also discuss the challenges of data sharing and privacy concerns, emphasizing the need for collaborative efforts and secure data practices to advance rare disease research.

## Introduction

Rare diseases pose significant challenges in diagnosis and treatment due to their low prevalence and diverse presentations. In the USA, a disease is considered rare if it affects fewer than 200,000 people, while in Europe, it is classified as rare if it affects fewer than 1 in 2000 individuals [1]. Despite their rarity, over 10,000 distinct types of rare and genetic diseases collectively affect around 400 million people globally [1]. Approximately, 80% of rare diseases are attributed to genetic causes, highlighting the importance of genetic testing for accurate diagnosis [1]. Understanding their genetic interplay has driven the development of targeted treatments such as gene therapy, gene editing, and personalized medicine.

Although big data has been integrated in rare disease genomics, major barriers still need to be addressed, including difficulties in identifying causal variants and translating findings into clinical practice. Various large consortia have increasingly emerged in response to these challenges, such as the National Biobank of Korea [2, 3], which contains the latest established large Korean rare disease cohort. The integration of AI and ML in rare disease research has improved the identification of disease-causing variants and enhanced diagnostic accuracy [4]. These technologies are driving advancements in precision medicine, enabling more personalized and effective treatments through gene-targeted therapies [5]. Data privacy concerns are inevitable in handling genomics data, and several efforts have been made to prevent the exposure of patient information, which will be explored further in this review. Hence, this paper aims to comprehensively review genomics techniques and tools used in rare disease research alongside therapeutic applications. Unlike previous review articles that have dealt with certain topics (e.g., deep learning or public health) [6, 7], this review will offer insights into the broader landscape of rare disease genomics and therapeutic medicine.

### Advancements in genomic technologies for rare disease diagnosis

Diagnosing rare diseases has been historically challenging. In the late twentieth century, Sanger sequencing was the most commonly used technique for about 25 years [8]. However, it could only analyze one gene at a time, making it time-consuming and costly, especially in cases involving genetic heterogeneity or unclear clinical manifestations [9–12].

The advent of next-generation sequencing (NGS) about a decade ago revolutionized the diagnostic workflow. Short-read sequencing (SRS) technologies, such as exome and genome sequencing, became incorporated into routine diagnostic procedures for rare diseases [13–16]. Whole exome sequencing (WES) has been applied to patients suspected of rare diseases with unusual phenotypic characteristics (e.g., cerebellar hypoplasia, epilepsy, or global developmental delay), leading to a definitive diagnosis for 28.3% of the patients [17]. However, due to the complicated genetic underpinnings of rare diseases, NGS-based methods had a detection rate of only 25–50% in undiagnosed patients [15].

To mitigate these limitations, long-read sequencing (LRS) has emerged as a promising tool, allowing for more accurate detection of complex genetic variants such as short tandem repeats (STRs), copy number variations (CNVs), and structural variants (SVs). Two primary LRS technologies have gained prominence: Oxford Nanopore Technologies’ nanopore sequencing and Pacific Biosciences’ (PacBio) single-molecule, real-time (SMRT) sequencing [18]. Both technologies offer advantages in detecting complex genetic variants but differ in approach and output characteristics.

Specifically, LRS has proven successful in diagnosing previously undiagnosed rare disease patients. For instance, nanopore LRS facilitated the detection of deep intronic variants in the TSC1 and TSC2 genes, leading to the identification of aberrant splicing events and a confirmed diagnosis of tuberous sclerosis [19]. Similarly, LRS enabled the diagnosis of patients with Cornelia de Lange syndrome (CDLS) by identifying a complex chromothripsis event affecting the NIPBL gene, which had been undetectable by SRS [20]. Furthermore, PacBio HiFi reads revealed a repeat expansion in the DAB1 gene, associated with spinocerebellar ataxia 37 (SCA37), in a family exhibiting autosomal dominant ataxia [21].

These cases demonstrate the utility of LRS in resolving diagnostically challenging genetic variants, particularly complex structural variants and intronic mutations, contributing significantly to the diagnosis of rare diseases.

### Collaborative efforts through large consortia

The establishment of large consortia for rare diseases addresses the need for coordinated research efforts [22]. Despite initiatives like the Rare Disease Clinical Research Network (RDCRN), rare disease research often remains siloed, focusing on single conditions [23]. In response, diverse collaborations have been launched to unite researchers and foster collaborative efforts across multiple rare diseases (Table 1).

**Table 1:** Overview of large consortia/initiatives for rare diseases

Consortia/initiatives	Description	Data availability	URL	Accession number	References
Global scale					
European Joint Programme on Rare Disease (EJP RD)	Europe-wide initiative with the aim of improving diagnosis and treatment of rare diseases	Data available within the website	https://resourcemap.ejprarediseases.org/	None	[24]
International Rare Diseases Research Consortium (IRDiRC)	Global Consortium that coordinates research efforts to develop 1000 new therapies for rare diseases by 2027	Data available within the website	https://irdirc.org/resources-2/irdirc-recognized-resources/	None	[25]
National Organization for Rare Disorders (NORD)	Advocacy organization in the USA that provides support for patients and advocates for rare disease research	Data available on request from the team	https://rarediseases.org/resource-library/	None	[26]
Rare Disease Clinical Research Network (RDCRN)	International collaboration designed to develop medical research on rare diseases with increased support for clinical studies	Data available on request from the team	https://www.rarediseasesnetwork.org/research/data-sharing-and-standards/data-sharing-resources	None	[27]
Undiagnosed Diseases Network International (UDNI)	Global network focused on enhancing the understanding of diagnosis of previously undiagnosed diseases	Data not publicly available	None	None	[28]
National scale					
Canadian Organization for Rare Disorders (CORD)	Advocacy organization in Canada focused on reinforcing public policy and support for the well-being of patients with rare diseases	Data not publicly available	None	None	[29]
CIHR Rare Disease Research Initiative	Canadian program under the Canadian Institutes of Health Research found to increase collaboration across the rare disease community	Data not publicly available	None	None	[30]
Initiative on Rare and Undiagnosed Diseases in Japan	National program in Japan dedicated to advancing research and healthcare strategies for rare diseases	Data not publicly available	None	None	[31]
Korean Undiagnosed Diseases Program (KUDP)	National program in South Korea aimed at diagnosing undiagnosed patients and building long-term research infrastructure	Data available on request from the authors	None	None	[32]

For example, task forces (TFs) [33], adopted by the International Rare Diseases Research Consortium (IRDiRC), have addressed actionable subjects such as reducing the duration of the diagnostic process [34]. The Matchmaker Exchange (MME) TF devised a federated platform to expedite gene discovery for rare diseases by matchmaking patients with similar phenotypes. Six novel candidate genes associated with rare diseases, including armfield X-linked intellectual disability (XLID) syndrome [35], neurodevelopmental disorder [36], polyneuropathy [37], and ZNFX1 deficiency [38], were identified from undiagnosed patients enrolled in Care4Rare Canada [39] through the application of MME. These consortia function as hubs for data exchange among researchers studying rare diseases.

### Big data analytics in rare disease genomics

Due to the implementation of large consortia, a lot of data, so-called big data, is accumulated, emphasizing the necessity of implementing a big data-based analysis pipeline. Processing big data presents impediments, including storage limitations, computational power requirements, and data security concerns [40]. Cloud platforms offer a scalable solution, enabling researchers to store and analyze large datasets efficiently [41]. Cloud platforms facilitate data sharing and collaboration without geographic constraints.

Researchers have increasingly utilized cloud platforms to analyze big data in rare diseases. For example, the All of Us Research Program utilizes a cloud-based Researcher Workbench built on Google Cloud through Terra, which provides secure computational power for analysis [42]. The Genome Analysis Toolkit (GATK) team recommended running GATK across various cloud platforms, particularly Terra, for its user-friendly graphical interface [43]. Amazon Web Services (AWS) hosts large public datasets, such as Genome Aggregation Database (gnomAD) [44], UK Biobank [45–47], and 100,000 Genomes Project (100KGP) [48] allowing users to analyze data and build services using a broad range of data analytics products.

DRAGEN is now widely available on platforms like Illumina Connected Analytics (ICA) and AWS Marketplace. It offers faster analysis times, requires fewer computational resources, and accurately detects various variants [49, 50]. For instance, while using BWA and HaplotypeCaller for variant calling requires 32 h, leveraging DRAGEN can significantly reduce this time to just 37 min [51]. Both methods show comparable accuracy in variant calling, with DRAGEN achieving 99.07% for single-nucleotide polymorphisms (SNPs) and 88.39% for insertions and deletions (indels), while Burrows-Wheeler Aligner (BWA) combined with HaplotypeCaller reaches 98.68% for SNPs and 89.45% for indels [51]. When analyzing large-scale data, cloud platforms and pipelines should be tailored to fit the user’s specific data and cost requirements [52].

### Artificial intelligence (AI) and machine learning (ML) in rare disease analysis

Patients with rare diseases often face challenges such as diagnostic delay and misdiagnosis, and more than 90% of rare diseases lack effective treatments [53–55]. AI and ML technologies contribute to rare disease research by assisting the analysis of vast amounts of genomic and clinical data to identify disease patterns, predict treatment outcomes, and develop personalized therapies, ultimately improving diagnostic accuracy and advancing drug development [56].

In the variant calling stage, deep learning models such as DeepVariant [57] and Clairvoyante [58] transform sequencing data into an image-like format and use convolutional neural networks (CNNs) to interpret DNA alignments as visual patterns for detecting genetic variants. Tools like NeoMutate [59], which utilize Bayesian classifiers and supervised learning algorithms, further integrate multiple methods to improve variant detection. These tools allow researchers to identify genetic variations with increased accuracy. DeepSVFilter [60], a CNN-based tool, filters SVs from genome sequencing data in the variant filtering stage. Tools like Intelli-NGS [61] use deep neural networks (DNNs) to minimize false-positive and false-negative rates, significantly improving the filtering process.

Once variants are identified, AI-driven tools aid in its annotation and prioritization. MetaSVM [62] and MetaLR [62] provide ensemble predictions for deleterious effects, while combined annotation-dependent depletion (CADD) [63] combines functional annotations and evolutionary conservation. Sorting Intolerant From Tolerant (SIFT) [64] and Polymorphism Phenotyping v2 (PolyPhen-2) [65] assess sequence homology and structural features, respectively. Variant Effect Scoring Tool (VEST3) [66] and Protein Variation Effect Analyzer (PROVEAN) [67] score the functional impact of missense mutations, and MutationTaster2 [68] incorporates evolutionary conservation and disease associations. Mendelian Clinically Applicable Pathogenicity (M-CAP) [69] classifies rare variants; Missense badness, PolyPhen-2, and Constraint (MPC) [70, 71] enhance predictions using constraint metrics, Functional Analysis through Hidden Markov Models with an eXtended Feature set (FATHMM-XF) [72], and Missense Variant Pathogenicity prediction (MVP) [73] focuses on potentially pathogenic variants. Additional tools include Skyhawk [74], DANN [75], DeepSEA [76], exome Disease Variant Analysis (eDiva) [77], and RENOVO [78], utilizing neural networks and random forest to prioritize clinically relevant variants and assess noncoding or germline variants.

AI has significantly advanced the field of phenotype-genotype association, particularly in diagnosing rare diseases. DeepGestalt [79], which employs a deep CNN, analyzes facial images to distinguish between genetic subtypes, offering powerful diagnostic support. Deep PhenomeNET Variant Predictor (DeepPVP) [80], modeled by adopting DNN, prioritizes variants by integrating patient phenotype information, enhancing the identification of disease-causing variants. Xrare [81] focuses on prioritizing causative gene variants in rare diseases by utilizing phenotype-genotype association methods, providing clinicians with a streamlined approach to diagnosis. Additionally, Super-quick Information content Random Forest Learning of Splice Variants (SQUIRLS) [82], which uses a random forest algorithm, classifies splice variants, further improving the genotype–phenotype correlation by assessing the impact of genetic variants on splicing mechanisms. These tools collectively enhance the accuracy and efficiency of rare disease diagnosis by linking phenotypic features with underlying genetic data. The integration of AI technologies with biomarker discovery from genomics data and advanced imaging diagnostics offers a promising approach to accelerating the diagnosis and treatment of rare diseases and reducing patients’ diagnostic odyssey. Additionally, the widespread implementation of AI-driven tools increases accessibility. It provides more comprehensive, data-driven insights, empowering clinicians and nonspecialists to make more informed decisions in managing rare genetic diseases.

### Expanding genomic research: perspectives from Korean Bio-Big Data

Despite representing about 22% of the global population, East Asians are under-represented in genetic research and are often missing from control databases. To address this imbalance, initiatives have been promoted to create a comprehensive Korean control database and to analyze the Korean Reference Genome.

Existing Korean databases include the Korean National Standard Reference Variome (KoVariome) [83], the Korean Reference Genome Database (KRGDB) [84], KOVA 2 [85, 86], the Korean Reference Genome (KRG), the Korean Genetic Diagnosis Program for Rare Diseases (KGDP), Korea4K [87], and National Biobank of Korea (Table 2) [2, 3]. KoVariome offers a comprehensive catalogue of genetic variations, including novel variants, enhancing the accuracy of identifying pathogenic genetic variants specific to the Korean population [83]. KRGDB contains genomic variant data, including frequency information, functional annotations, and genome-wide association studies (GWAS) results for common diseases [84]. The KOVA 2, built on the earlier KOVA dataset [85], offers critical insights into population-specific genetic variants and loci under selection [85, 86].

**Table 2:** Major databases of Korean Bio Big Data

Database	No. of individuals	Technology	Sample type	Published year	Data availability	URL	Accession number	Reference
KoVariome	50	WGS	Healthy individuals	2018	Data available within the article or its supplementary materials	https://koreangenome.org/The_Korean_Reference_Variome:_KoVariome	None (uses FTP server)	Kim et al. [83]
KRGDB	1722	WGS	Integrated	2020	Data available on request from the authors	None	None	Jung et al. [84]
KOVA 2	1896	WGS	Healthy individuals	2022	Data available within the article or its supplementary materials	https://www.kobic.re.kr/kova/downloads	None (uses FTP server)	Lee et al. [86]
	3409	WES						
KRG (pilot phase)	1490	WGS	Healthy individuals	2022	Data available on request from the authors	None	None	Hwang et al. [88]
KGDP (Phase II)	1890	Multi-omics	Rare disease patients	2023	Data available on request from the authors	None	None	Kim et al. [89]
Korea4K	4157	WGS	Integrated	2024	Data available on request from the authors	https://ega-archive.org/studies/EGAS00001007580	EGAD00001015348	Jeon et al. [87]
National Biobank of Korea (pilot phase)	772,319	Multi-omics	Integrated	2024	Data generated at a National Biobank of Korea, available upon request	https://biobank.nih.go.kr/cadaver/EgovPageLink.do?menuNo=34&link=eng%2Fmain%2Fcontent%2FBiobankingActives%2FControlPage	None	[2, 3]
	14,905	WGS	Rare diseases patients and their relatives					

As of September 2024

KRG project aims to identify the genome architecture of the Korean population and develop Korean-specific genomic resources, intending to include 20,000 participants [88]. KGDP Phase II enhances diagnostic capabilities through collaboration with the Korean Undiagnosed Diseases Program (KUDP) [89, 90]. In 2024, Jeon et al. presented the second phase of the Korean Genome Project (KGP), known as Korea4K, to build a comprehensive reference dataset [87]. Korea4K provides a valuable large-scale genome-phenome variome database for the Korean population and detailed information on various clinical traits, representing the most extensive genomic and phenomic data resources [87]. Beyond control databases, the rare disease cohort in the National Biobank of Korea Project includes whole genome sequencing (WGS) data from 14,905 patients in a pilot study, aiming to expand to a cohort of 400,000 by 2028. A pilot study on this rare disease cohort enables estimation of neuronal intranuclear inclusion disease (NIID) prevalence in the Korean population [91].

### Strategies for identifying and characterizing pathogenic variants

**Fig. 1:**
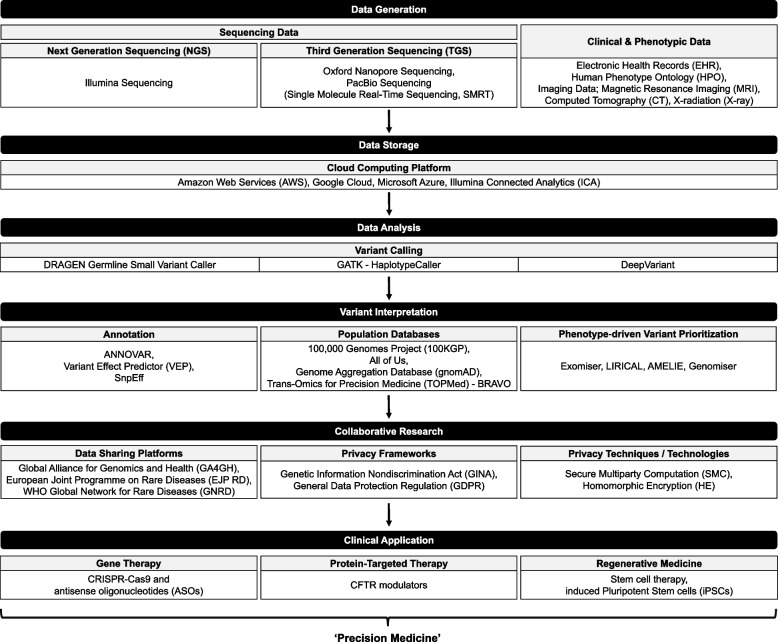
Integrated workflow for rare disease diagnosis and research

The process of data acquisition, identifying and characterizing genetic variants, followed by clinical application, involves multiple steps (Fig. 1). While single-nucleotide variant and small insertion and deletion variant calling has been robust along with the development of variant calling tools like GATK, DRAGEN, and DeepVariant, interpreting variants’ pathogenicity and their relevance to specific phenotypes remains challenging [57]. Annotation databases such as ANNOVAR, Variant Effect Predictor (VEP), and SnpEff [92] are publicly available for research. Still, the sheer volume of data and variability in clinical significance complicate the interpretation process [93, 94].

At the variant level, despite the availability of numerous tools for predicting the pathogenicity of missense variants [95], accurately determining the clinical significance of these variants remains a significant challenge in genomic interpretation [72, 96–98]. Deep learning models like AlphaMissense and PrimateAI-3D have recently been developed to predict variants’ pathogenicity [99, 100]. AlphaMissense utilizes AlphaFold’s structural predictions and evolutionary conservation to achieve 90% precision on the ClinVar dataset [101], excelling in identifying deleterious variants in conserved regions and correlating well with multiplexed assays of variant effect (MAVEs) data [99, 102]. PrimateAI-3D outperforms AlphaMissense in real-world cohorts, including rare disease patients with clinical characteristics, including developmental disorders (DDD), autism spectrum disorders (ASD), and congenital heart disorders (CHD). It shows superior predictive power in biobank phenotypes and proteomics [103].

Another essential aspect of variant characterization and interpretation is the frequency of variants. Large population databases such as the gnomAD and NHLBI’s Trans-Omics for Precision Medicine (TOPMed)-BRAVO help researchers determine how rare a variant is [70, 104]. Rare variants are frequently linked to rare diseases due to their potential to disrupt critical biological functions or pathways essential for health. Their low frequency in the general population often reflects negative selection effects, as highly pathogenic variants tend to be eliminated from the gene pool over time due to their detrimental impact on reproductive fitness.

For instance, the identification of a novel variant in the NSD1 gene, which has been reported to occur at a low allele frequency (MAF = 0.006%, 7/114,570) in gnomAD3.1.1, has provided valuable insights into its potential pathogenic role in patients with Sotos syndrome [105]. However, common variants also play a role in rare disease etiology as genetic modifiers influencing disease onset, progression, or severity [106]. In this context, polygenic risk scores (PRS), which aggregate the effects of many common variants, are increasingly being explored in rare disease genetics to help explain variable expressivity and incomplete penetrance and to potentially improve diagnostic and prognostic accuracy in conjunction with rare variant analysis [107]. For instance, the study examined 2759 cases with developmental and epileptic encephalopathies (DEEs) or epilepsy with intellectual disability (ID) and 447,760 population-matched controls to explore the relevance of PRS [108]. It found that even in cases with a known deleterious variant, common genetic variation contributes significantly to the risk, explaining between 0.08 and 3.3% of the phenotypic variance across epilepsy subtypes [108].

The landscape of rare disease genetics has evolved significantly with the advent of WGS. While historically focused on exonic mutations, research now recognizes the importance of noncoding regions in harboring disease-causing variants [109, 110]. However, accurately classifying these noncoding variants remains challenging. Current guidelines, such as those from American College of Medical Genetics and Genomics (ACMG) and the Association for Molecular Pathology (AMP), primarily address coding variants [111], leaving a gap in interpreting noncoding variants [112]. To address this, new recommendations have emerged, focusing on defining regulatory regions, filtering clinically relevant variants, incorporating functional evidence (e.g., RNA sequencing, chromatin interaction assays), and applying bioinformatics tools like SpliceAI [113], MotifbreakR [114], and UTRannotator [115] to assess their pathogenicity [112]. These approaches aim to provide a more comprehensive framework for evaluating variants across the entire genome, potentially enhancing rare disease diagnosis and understanding.

Finding the causal variant of rare diseases necessitates precise evaluation and prioritization of genetic variants. Previous prioritization methods have primarily focused on in silico assessments of variant pathogenicity, resulting in decreased sensitivity and difficulties in understanding the results. While valuable, manual curation of genetic variants is limited by human error, subjectivity, and the overwhelming volume of data produced by NGS technologies. These biases can lead to missed or incorrectly prioritized variants, particularly in noncoding regions or when dealing with novel variants lacking extensive annotation. Automated gene/variant prioritization tools such as Exomiser [116], MAVERICK [117], LIkelihood Ratio Interpretation of Clinical AbnormaLities (LIRICAL) [118], Automatic Mendelian Literature Evaluation (AMELIE) [119], and Genomiser [120] significantly reduce manual curation efforts and minimize human bias in rare disease diagnosis. These tools integrate diverse information sources to generate a ranked list of candidate causal genes or variants, including phenotypic data encoded as Human Phenotype Ontology (HPO) terms, known disease associations, and functional predictions [118, 119]. By systematically and exhaustively analyzing vast amounts of data, these resources provide a comprehensive and unbiased approach to variant interpretation, surpassing the limitations of manual literature searches. This automation improves diagnostic precision and efficiency and enables more consistent and reproducible results across different clinical settings. Consequently, these tools enhance treatment strategies and patient outcomes in precision medicine, offering a scalable solution to the growing complexity of genomic interpretation in rare disease diagnostics.

### Therapeutic innovations and precision medicine approaches

Therapeutic implications and precision medicine for rare diseases increasingly rely on advanced genomic technologies like WES and WGS. These tools enable the identification of pathogenic variants, allowing for tailored treatment strategies. Gene therapies, such as clustered regularly interspaced short palindromic repeats (CRISPR)-Cas9 and antisense oligonucleotides (ASOs), are at the forefront of this approach. For example, onasemnogene abeparvovec (Zolgensma) treats spinal muscular atrophy (SMA) by delivering a functional SMN1 gene [121], while nusinersen (Spinraza) modifies SMN2 splicing to enhance functional protein levels [122].

Protein-targeted therapies, like CFTR modulators for cystic fibrosis, improve defective protein function directly [123]. Recent advancements in regenerative medicine, including stem cell therapy and induced pluripotent stem cells (iPSCs), also offer promising avenues for repairing damaged tissues [124]. Together, these innovative strategies enhance patient outcomes and demonstrate the potential of precision medicine in rare disease treatment.

### Challenges in data sharing and privacy concerns

Data sharing between researchers is essential in advancing rare disease research, as it increases diagnostic yield and unravels the underlying disease mechanisms. For instance, the German TRANSLATE-NAMSE project found that interdisciplinary case conferences led to definitive diagnoses for 32% of pediatric and 26% of adult patients previously undiagnosed [6, 125]. The Global Alliance for Genomics and Health (GA4GH), an international coalition with members from over 90 countries, was established to facilitate sharing of genomic and clinical data and promote interoperability among institutions.

The European Joint Programme on Rare Diseases (EJP RD), one of 24 ‘Driver Projects’ of GA4GH, maintains repositories containing more than 130,000 WES and WGS datasets across multiple resources including the European Genome-Phenome Archive (EGA), DECIPHER, and the RD-Connect Genome-Phenome Analysis Platform (GPAP) [126]. In 2023, EJP RD launched a Virtual Platform, a public portal that provides access to Findable, Accessible, Interoperable, and Reusable (FAIR)-compliant resources, streamlining data searching while safeguarding patient confidentiality [127]. International data exchange brings significant benefits.

However, data privacy remains a critical challenge, particularly for genomic and clinical data. Data misuse can violate the privacy of individuals and their biological relatives. Individual patients can be uniquely identified through distinctive genetic markers, such as rare single-nucleotide variants (SNVs) specific to their genome [128, 129].

To tackle this privacy concern, frameworks such as the Genetic Information Nondiscrimination Act of 2008 (GINA) and the General Data Protection Regulation (GDPR) [130, 131] have introduced frameworks ensuring data security. Despite these efforts, legal protections remain inconsistent, especially in the USA, where federal laws like HIPAA provide limited protection, particularly once data has been anonymized, as this anonymized data can be reidentified using several techniques, such as surname inference [132]. Some participants in the 100KGP were reidentified as their surnames could be inferred by analyzing Y-chromosome STRs and cross-referencing with genealogy databases [133].

There is an unavoidable trade-off between data privacy concerns and the societal benefits of data sharing. An approach to mitigate the risk of reidentification includes employing cryptographic methods, such as secure multiparty computation (SMC), to secure genomic data sharing and allow computations without exposing raw data. SMC enables multiple parties to jointly compute GWAS statistics, such as minor allele frequency, without sharing their raw data [134]. Ultimately, privacy-preserving strategies should be prioritized to ensure the benefits of data sharing in rare disease research do not come at the cost of individual privacy.

## Conclusion

The field of rare disease research has undergone significant advancements, driven by technological innovations in genomic sequencing, big data analytics, and AI. LRS technologies, cloud computing platforms, and AI/ML-driven tools have greatly enhanced our ability to detect complex genetic variants and interpret their clinical significance. Large-scale collaborative efforts and the establishment of comprehensive genomic databases have expanded our knowledge of rare diseases.

Although significant progress has been made, challenges continue to arise. The complexity of variant interpretation calls for advanced prediction tools and automated systems for prioritization. Additionally, while sharing data is crucial for further research, it introduces privacy concerns that must be addressed through robust legal frameworks and advanced privacy-preserving technologies.

The integration of multi-omics data, the refinement of AI models, and the expansion of diverse population databases will be vital in advancing the diagnosis and treatment of rare diseases. The emergence of precision medicine, mainly through gene and protein-targeted therapies, highlights its potential in rare disease management. As the field continues to balance collaborative data sharing with stringent privacy protections, significant progress is expected in understanding, diagnosing, and treating rare diseases, ultimately enhancing the lives of millions of affected individuals worldwide.

## Data Availability

No datasets were generated or analysed during the current study.
